# Molecular Hydrogen Reverses Nociplastic Pain and Depressive-like Behaviors via Region- and Sex-Dependent Central Mechanisms

**DOI:** 10.3390/ijms27073051

**Published:** 2026-03-27

**Authors:** Sylmara Esther Negrini-Ferrari, Ignacio Martínez-Martel, Olga Pol

**Affiliations:** 1 Grup de Neurofarmacologia Molecular, Institut de Recerca Sant Pau (IR SANT PAU), Sant Quintí 77-79, 08041 Barcelona, Spain; 2 Grup de Neurofarmacologia Molecular, Institut de Neurociències, Universitat Autònoma de Barcelona, 08193 Barcelona, Spain

**Keywords:** depression, fibromyalgia, hydrogen-rich water, inflammation, molecular hydrogen, nociplastic pain, oxidative stress

## Abstract

Fibromyalgia is a chronic nociplastic pain condition frequently accompanied by affective disturbances, particularly depression, for which effective treatments remain limited. Increasing evidence implicates central oxidative stress, maladaptive synaptic plasticity, and neuroinflammatory alterations in its pathophysiology. This study investigated the therapeutic effects of molecular hydrogen (H_2_) in a reserpine-induced murine model of fibromyalgia, with emphasis on sex-dependent and region-specific mechanisms. Male and female C57BL/6 mice received repeated reserpine injections to induce fibromyalgia-like symptoms. Mechanical allodynia, thermal hyperalgesia, cold allodynia, and depressive-like behaviors were assessed, followed by molecular analyses in the spinal cord and amygdala. Reserpine induced persistent nociceptive hypersensitivity and depressive-like behaviors in both sexes, with earlier cold allodynia in females. Hydrogen-rich water (HRW) progressively reversed mechanical and thermal hypersensitivity and rapidly abolished cold allodynia, showing greater efficacy in females. HRW also normalized depressive-like behaviors in both sexes. At the molecular level, HRW reduced spinal oxidative stress and ERK-dependent plasticity without altering spinal NLRP3 expression, whereas it fully reversed NLRP3 upregulation and HO-1 downregulation in the amygdala. HRW additionally engaged sex-dependent antioxidant pathways in the spinal cord. These findings indicate that H_2_ alleviates sensory and affective alterations through region- and sex-dependent central mechanisms, supporting HRW as a promising therapeutic strategy for nociplastic pain and its affective comorbidities.

## 1. Introduction

Fibromyalgia is a chronic syndrome characterized by widespread musculoskeletal pain, fatigue, and localized tenderness [1]. Despite its high prevalence and debilitating nature, its pathophysiology remains poorly understood [2]. Epidemiological studies estimate that fibromyalgia affects approximately 2–4% of the general population, with a markedly higher prevalence in women [3]. These assessments commonly include measurements of pain intensity, pressure pain threshold, sleep quality, anxiety, depression, and overall functional status [4,5]. Current therapeutic approaches provide limited symptom relief, underscoring the urgent need for the development of more effective pharmacological strategies.

In this context, growing evidence indicates that oxidative stress, mitochondrial dysfunction, and inflammation play a central role in the development and persistence of fibromyalgia symptoms [6]. Pro-oxidative processes have been closely associated with pain sensitization, particularly within the central nervous system, where lipid peroxidation contributes to elevated levels of oxidative stress biomarkers such as malondialdehyde and 4-hydroxynonenal (4-HNE) [1,7]. Moreover, mitochondrial dysfunction driven by excessive production of reactive oxygen species (ROS) has been linked to enhanced nociceptive signaling, potentially through disruptions in cellular energy metabolism and the facilitation of neuroinflammatory processes [8]. Consistent with this, an imbalance between ROS generation and antioxidant defense mechanisms represents a hallmark feature of fibromyalgia pathophysiology, as patients exhibit reduced total antioxidant capacity and decreased activity of key antioxidant enzymes, including superoxide dismutase (SOD), catalase, and glutathione peroxidase, which may exacerbate oxidative damage and contribute to pain amplification [9,10].

Furthermore, emerging evidence suggests that activation of the NOD-like receptor family pyrin domain-containing 3 (NLRP3) inflammasome may represent a shared mechanistic link between fibromyalgia and depression. In fibromyalgia, sustained NLRP3 inflammasome signaling activation contributes to chronic inflammation, mitochondrial dysfunction, and neuroimmune alterations that promote pain sensitization and mood dysregulation [11].

Pharmacological treatment remains a cornerstone of fibromyalgia management. Currently, gabapentinoids and antidepressants constitute the mainstay of pharmacotherapy, including agents such as pregabalin, duloxetine, and milnacipran. However, the therapeutic efficacy of these drugs varies considerably across clinical studies, with meaningful pain relief achieved in only approximately 30–60% of patients and often limited to a reduction of about 30%, alongside a significant burden of adverse effects [12]. These limitations underscore the need for continued research into more effective and better-tolerated therapeutic strategies.

Molecular hydrogen (H_2_) is emerging as a promising therapeutic strategy targeting oxidative stress, mitochondrial dysfunction, and inflammation, which are key contributors to fibromyalgia pathophysiology. As a selective antioxidant, H_2_ neutralizes highly reactive oxygen species such as hydroxyl radicals, a mechanism critically implicated in fibromyalgia, thereby restoring redox homeostasis [8]. Preclinical studies have demonstrated that hydrogen-rich water (HRW) attenuates allodynia and hyperalgesia in models of neuropathic pain induced by nerve injury or chemotherapy through the modulation of oxidative stress and inflammatory pathways. Specifically, HRW upregulates antioxidant enzymes such as heme oxygenase-1 (HO-1) and superoxide dismutase-1 (SOD-1), while suppressing pro-inflammatory cytokine production and nuclear factor kappa B activation [13,14]. In addition, HRW enhances mitochondrial function and ATP-sensitive potassium channel activity, contributing to its analgesic and neuroprotective effects [14,15]. Notably, HRW has also been shown to exert antidepressant-like effects in preclinical models, which may be particularly relevant given the high prevalence of mood disorders in fibromyalgia, and that the repeated administration of H_2_ has not been associated with adverse effects or tolerance to date [16]. Nonetheless, the potential of H_2_ to alleviate fibromyalgia-related pain particularly with consideration of sex as a biological variable and depressive-like behaviors remains largely unexplored.

In this study, we employed a well-established murine model of fibromyalgia induced by repeated administration of reserpine, which faithfully reproduces key neurochemical and behavioral features of the disorder [17,18]. Repeated reserpine treatment leads to depletion of monoaminergic neurotransmitters, including dopamine, serotonin, and norepinephrine, while simultaneously enhancing excitatory neurotransmission through increased levels of substance P and glutamate in the spinal cord and pain-processing brain regions such as the thalamus and prefrontal cortex [19,20,21]. As a consequence, animals subjected to repeated reserpine administration develop a phenotype that closely resembles the clinical features of fibromyalgia, including allodynia and hyperalgesia, depressive-like behaviors, and cognitive impairments [20,22]. Importantly, the oxidative stress, mitochondrial dysfunction, and neuroinflammatory alterations induced by reserpine further support the translational relevance of this model for mechanistic and pharmacological investigations [22,23,24].

Therefore, using this model, the aim of the present study was to investigate the therapeutic effects of HRW on fibromyalgia-related symptoms, including pain and depressive-like behaviors. We hypothesized that HRW alleviates fibromyalgia symptoms through the modulation of oxidative stress, inflammatory pathways, and/or synaptic plasticity alterations in the spinal cord and the amygdala, two key regions involved in pain processing and emotional regulation, respectively [25,26].

## 2. Results

### 2.1. Reserpine Induces Persistent Mechanical Allodynia, Thermal Hyperalgesia and Cold Allodynia in Male and Female Mice

**Figure 1:**
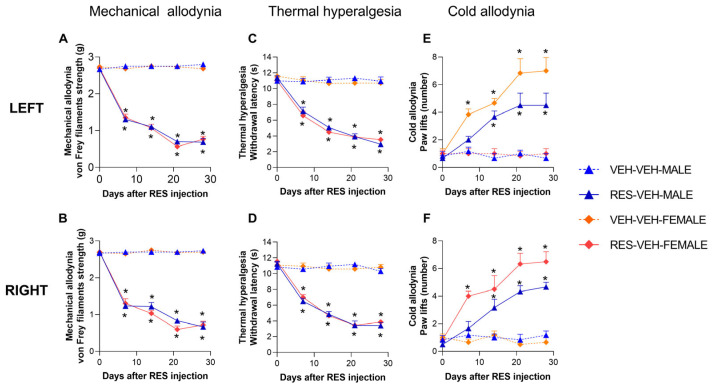
Reserpine-induced nociceptive hypersensitivity in male and female mice. Mechanical allodynia (**A**,**B**), thermal hyperalgesia (**C**,**D**), and cold allodynia (**E**,**F**) were evaluated in the left and right hind paws of male and female mice following reserpine administration. Mechanical allodynia is expressed as von Frey withdrawal threshold (g), thermal hyperalgesia as paw withdrawal latency (s), and cold allodynia as the number of paw lifts. Symbols denote significant differences compared with the corresponding VEH–VEH (*) group at each time point (*p <* 0.05; one-way ANOVA followed by Sidak’s post hoc test). Data are presented as mean ± SEM (*n* = 6 per group). RES, reserpine.

Hind paw withdrawal responses to mechanical and thermal stimulation were evaluated in reserpine-injected male and female mice at baseline (day 0) and at 7, 14, 21, and 28 days after the first reserpine injection. The effects of treatment, sex, and time on mechanical allodynia and thermal hyperalgesia were analyzed in both the right and left hind paws. For both tests, three-way repeated-measures ANOVA revealed significant main effects of treatment (*p <* 0.0001) and time (*p <* 0.0001), as well as a significant treatment × time interaction (*p <* 0.0001), in both paws of male and female mice. Accordingly, reserpine administration produced a comparable reduction in hind paw withdrawal thresholds to von Frey filament stimulation (Figure 1A,B) and radiant heat stimulation (Figure 1C,D) at days 7, 14, 21, and 28 post-injections in both sexes (*p <* 0.001, one-way ANOVA vs. corresponding vehicle-treated controls).

These results further revealed that reserpine administration also induced cold allodynia with a sex-dependent temporal profile. Three-way repeated-measures ANOVA showed significant main effects of treatment (*p <* 0.0001), sex (*p <* 0.0031), and time (*p <* 0.0001), as well as significant treatment × sex (*p <* 0.0250) and treatment × time (*p <* 0.0001) interactions in both hind paws. Reserpine induced cold allodynia in both male and female mice, as evidenced by an increased number of paw lifts in response to cold stimulation in the left (Figure 1E) and right (Figure 1F) paws compared with vehicle-treated controls. Cold allodynia was observed at days 7, 14, 21, and 28 post-injections in female mice (*p <* 0.0001, one-way ANOVA) and at days 14, 21, and 28 in male mice (*p <* 0.0001, one-way ANOVA).

### 2.2. HRW Reverses Reserpine-Induced Nociceptive Hypersensitivity

Animals received HRW (0.3 mM) or vehicle intraperitoneally injected once daily for six consecutive days (days 23–28 after the first reserpine injection), a time point at which nociceptive hypersensitivity is fully established in this model.

Three-way repeated-measures ANOVA revealed significant main effects of treatment (*p <* 0.0001) and time (*p <* 0.0001), as well as a significant treatment × time interaction (*p <* 0.0001), for reserpine-induced mechanical allodynia, thermal hyperalgesia, and cold allodynia. In addition, a significant treatment × sex interaction was detected for cold allodynia in the cold plate test (*p <* 0.008).

**Figure 2:**
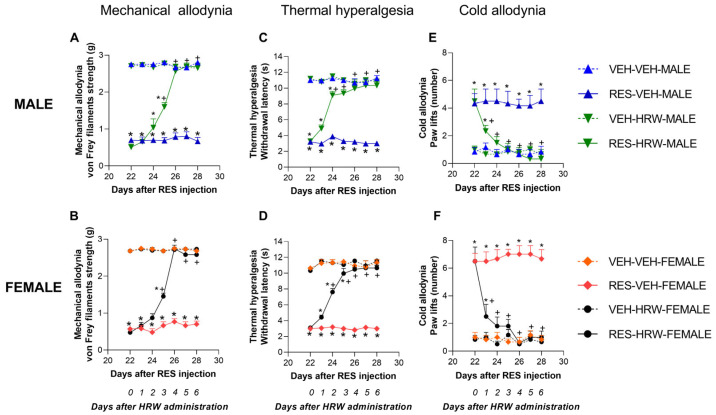
HRW attenuates reserpine-induced nociceptive hypersensitivity in male and female mice. The effects of HRW on mechanical allodynia (**A**,**B**), thermal hyperalgesia (**C**,**D**), and cold allodynia (**E**,**F**) were assessed in the hind paws of reserpine-injected male and female mice. Mechanical allodynia is expressed as von Frey withdrawal threshold (g), thermal hyperalgesia as paw withdrawal latency (s), and cold allodynia as the number of paw lifts. Symbols denote significant differences compared with the corresponding VEH–VEH (*) and RES–VEH (+) groups at each time point (*p <* 0.05; one-way ANOVA followed by Sidak’s post hoc test). Data are presented as mean ± SEM (*n* = 6 per group). RES, reserpine.

HRW administration progressively attenuated reserpine-induced mechanical allodynia over the course of treatment in both male and female mice. A comparable temporal pattern was observed in males (*p <* 0.0001; one-way ANOVA; Figure 2A) and females (*p <* 0.0001; one-way ANOVA; Figure 2B), with complete reversal of mechanical allodynia achieved after four days of HRW treatment in both sexes. Similar effects were observed for thermal hyperalgesia, although the time required for full reversal differed between sexes. In male mice, thermal hyperalgesia was fully reversed after three days of HRW treatment (*p <* 0.0001; one-way ANOVA; Figure 2C), whereas in female mice complete reversal was observed after four days of treatment (*p <* 0.0001; one-way ANOVA; Figure 2D).

HRW administration also reduced the increased number of paw lifts induced by reserpine in the cold plate test. In this case, a single day of HRW treatment was sufficient to abolish reserpine-induced cold allodynia in both male (*p <* 0.0001; one-way ANOVA; Figure 2E) and female mice (*p <* 0.0001; one-way ANOVA; Figure 2F). Notably, the magnitude of inhibition produced by HRW after one day of treatment was greater in female mice (61.5%) than in males (48.9%), consistent with the significant treatment × sex interaction observed in this test.

Comparable results were obtained in the right hind paws of reserpine-injected mice of both sexes across all paradigms, and HRW had no effect on either hind paw in VEH-treated mice.

### 2.3. Treatment with HRW Inhibited the Depressive-like Behaviors Associated with Fibromyalgia

Depressive-like behaviors were evaluated after six days of HRW treatment (day 28 post-reserpine injection). Our data confirmed the presence of depressive-like behaviors previously described in female reserpine-injected mice and further demonstrated that these emotional alterations also occur in male mice, as assessed by TST and FST. Three-way ANOVA revealed significant main effects of injection (*p <* 0.0001) and treatment (*p <* 0.0001), as well as a significant injection × treatment interaction (*p <* 0.0001), in both the TST and FST. In contrast, no significant effect of sex or interactions involving sex was detected in either test.

**Figure 3:**
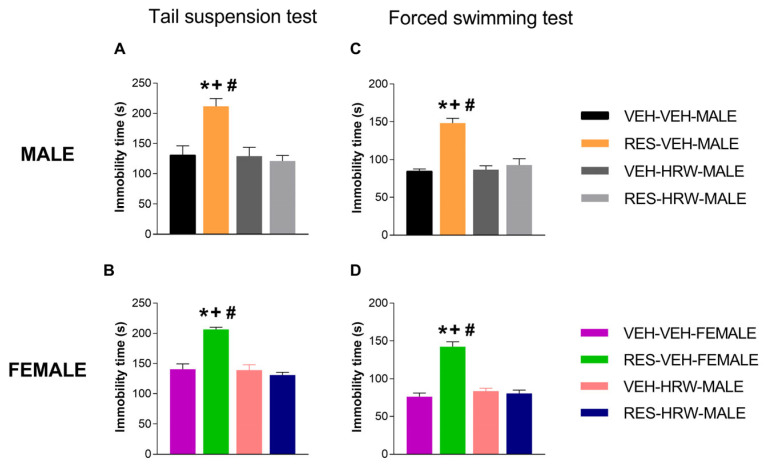
HRW reverses reserpine-induced depressive-like behaviors in male and female mice. The effects of HRW on immobility time in the tail suspension test (TST; (**A**,**B**)) and forced swimming test (FST; (**C**,**D**)) were evaluated in male and female mice injected with reserpine or vehicle. Behavioral assessments were performed after six consecutive days of HRW treatment. Symbols denote significant differences compared with the corresponding VEH–VEH (*), VEH–HRW (+), and RES–HRW (#) groups (*p <* 0.05; one-way ANOVA followed by Sidak’s post hoc test). Data are presented as mean ± SEM (*n* = 6 per group). RES, reserpine.

Accordingly, reserpine administration significantly increased immobility time in both male and female mice in the TST (*p <* 0.001; one-way ANOVA; Figure 3A,B) and the FST (*p <* 0.001; one-way ANOVA; Figure 3C,D). Treatment with HRW effectively normalized the increased immobility time in both sexes across the two behavioral paradigms. In contrast, HRW administration did not alter immobility time in VEH-treated mice of either sex in either the TST or FST.

### 2.4. The Effect of HRW Treatment on the Expression of 4-HNE, NLRP3 and p-ERK 1/2 in the Spinal Cord of Male and Female Reserpine-Injected Mice

**Figure 4:**
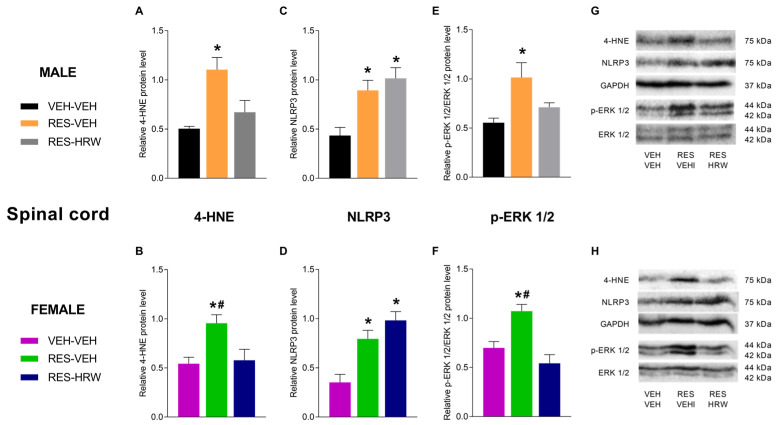
HRW modulates oxidative stress, inflammasome activation, and plasticity-related signaling in the spinal cord of reserpine-injected male and female mice. The effects of HRW on spinal protein levels of 4-hydroxynonenal (4-HNE; (**A**,**B**)), NLRP3 (**C**,**D**), and phosphorylated ERK1/2 (p-ERK1/2; (**E**,**F**)) were analyzed in male and female mice injected with reserpine and treated with VEH or HRW. For both sexes, VEH-injected mice treated with VEH are shown as controls. Protein levels of 4-HNE and NLRP3 are expressed relative to GAPDH, and p-ERK1/2 is expressed relative to total ERK1/2. Representative immunoblots for 4-HNE, NLRP3, GAPDH, p-ERK1/2, and ERK1/2 from male (**G**) and female (**H**) mice are shown. Symbols denote significant differences compared with the corresponding VEH–VEH (*), and RES–HRW (#) groups (*p <* 0.05; one-way ANOVA followed by Sidak’s post hoc test). Tissue samples were collected after six days of HRW treatment (day 28 post-reserpine injection). Data are presented as mean ± SEM (*n* = 4 samples per group). RES, reserpine.

To explore the molecular mechanisms underlying the effects of HRW in male and female reserpine-injected mice, protein levels of 4-HNE, NLRP3, and p-ERK1/2 were analyzed in the spinal cord. Two-way ANOVA revealed a significant main effect of treatment (*p <* 0.0001), with no significant effects of sex or treatment × sex interaction, on the expression of 4-HNE, NLRP3, and p-ERK1/2. Accordingly, reserpine significantly increased spinal cord 4-HNE levels in both male (*p <* 0.0135; one-way ANOVA vs. VEH; Figure 4A) and female mice (*p <* 0.0093; one-way ANOVA vs. VEH; Figure 4B). In both sexes, HRW treatment normalized the reserpine-induced increase in 4-HNE expression.

Consistent with the involvement of inflammatory mechanisms, reserpine administration significantly increased NLRP3 protein levels in the spinal cord of both male (*p <* 0.0030; one-way ANOVA vs. VEH; Figure 4C) and female mice (*p <* 0.0013; one-way ANOVA vs. VEH; Figure 4D). Notably, elevated NLRP3 levels persisted in HRW-treated animals of both sexes.

Regarding p-ERK1/2, reserpine administration significantly increased its expression in the spinal cord of both male (*p <* 0.0141; one-way ANOVA vs. VEH; Figure 4E) and female mice (*p <* 0.0005; one-way ANOVA vs. VEH; Figure 4F), indicating reserpine-induced plasticity changes in both sexes. Remarkably, HRW treatment normalized the elevated spinal cord levels of p-ERK1/2 in male and female mice.

### 2.5. The Effect of HRW Treatment on the Expression of HO-1, NQO1 and SOD-1 in the Spinal Cord of Male and Female Reserpine-Injected Mice

**Figure 5:**
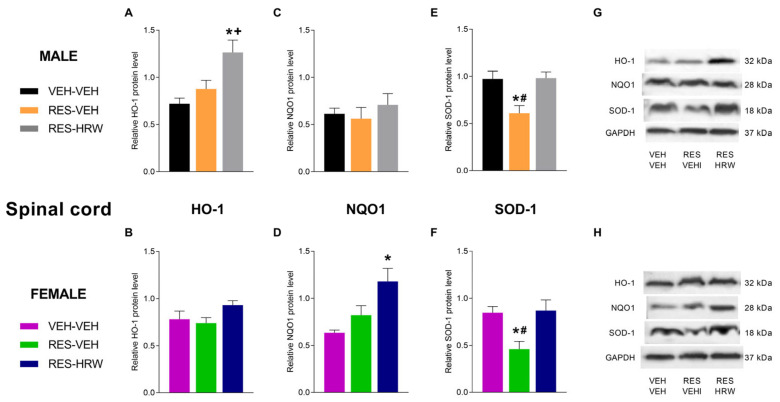
Sex-dependent modulation of spinal antioxidant enzymes by HRW in reserpine-injected mice. The effects of HRW on spinal protein levels of heme oxygenase-1 (HO-1; (**A**,**B**)), NAD(P)H quinone dehydrogenase 1 (NQO1; (**C**,**D**)), and superoxide dismutase 1 (SOD-1; (**E**,**F**)) were evaluated in male and female mice injected with reserpine and treated with VEH or HRW. For both sexes, VEH-injected mice treated with VEH are shown as controls. All protein levels are expressed relative to GAPDH. Representative immunoblots for HO-1, NQO1, SOD-1, and GAPDH from male (**G**) and female (**H**) mice are shown. Symbols denote significant differences compared with the corresponding VEH–VEH (*), RES-VEH (+), and RES–HRW (#) groups (*p <* 0.05; one-way ANOVA followed by Sidak’s post hoc test). Tissue samples were collected after six days of HRW treatment (day 28 post-reserpine injection). Data are presented as mean ± SEM (*n* = 4 samples per group). RES, reserpine.

Protein levels of the antioxidant enzymes HO-1, NQO1, and SOD-1 were evaluated in the spinal cord of male and female mice. Two-way ANOVA revealed significant main effects of sex (*p <* 0.0450) and treatment (*p <* 0.0001) on HO-1 expression. While reserpine did not modify HO-1 levels in either sex, HRW treatment significantly increased HO-1 expression in male mice (*p <* 0.0018; one-way ANOVA vs. VEH and RES; Figure 5A), but not in females (Figure 5B).

For NQO1, two-way ANOVA showed significant effects of sex (*p <* 0.0100) and treatment (*p <* 0.0170). Reserpine did not alter NQO1 expression in male mice (Figure 5C), whereas a significant increase was observed in female mice following HRW treatment (*p <* 0.0112; one-way ANOVA vs. VEH and RES; Figure 5D).

Regarding SOD-1, two-way ANOVA revealed a significant main effect of treatment only (*p <* 0.0001). Reserpine injection significantly reduced SOD-1 expression in both male (*p* < 0.0064; one-way ANOVA vs. VEH; Figure 5E) and female mice (*p* < 0.0054; one-way ANOVA vs. VEH; Figure 5F). In both sexes, HRW treatment normalized the reserpine-induced downregulation of SOD-1.

### 2.6. The Effect of HRW Treatment on the Expression of 4-HNE, NLRP3 and p-ERK 1/2 in the Amygdala of Male and Female Reserpine-Injected Mice

**Figure 6:**
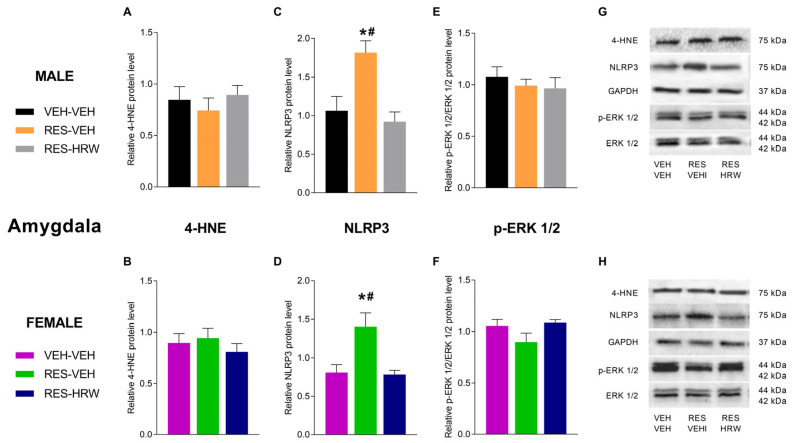
HRW differentially regulates oxidative stress, inflammasome activation, and plasticity-related signaling in the amygdala of reserpine-injected male and female mice. The effects of HRW on the protein levels of 4-hydroxynonenal (4-HNE; (**A**,**B**)), NLRP3 (**C**,**D**), and phosphorylated ERK1/2 (p-ERK1/2; (**E**,**F**)) in the amygdala were analyzed in male and female mice injected with reserpine and treated with VEH or HRW. For both sexes, VEH-injected mice treated with VEH are shown as controls. Protein levels of 4-HNE and NLRP3 are expressed relative to GAPDH, and p-ERK1/2 is expressed relative to total ERK1/2. Representative immunoblots for 4-HNE, NLRP3, GAPDH, p-ERK1/2, and ERK1/2 from male (**G**) and female (**H**) mice are shown. Symbols denote significant differences compared with the corresponding VEH–VEH (*) and RES–HRW (#) groups (*p <* 0.05; one-way ANOVA followed by Sidak’s post hoc test). Tissue samples were collected after six days of HRW treatment (day 28 post-reserpine injection). Data are presented as mean ± SEM (*n* = 4 samples per group). RES, reserpine.

Given that reserpine-injected mice exhibited depressive-like behaviors, we examined whether HRW treatment modulated reserpine-induced oxidative stress, inflammatory signaling, and plasticity-related alterations in the amygdala. Two-way ANOVA revealed no significant main effects of sex, treatment, or their interaction on 4-HNE or p-ERK1/2 expression in this brain region. In contrast, a significant main effect of sex (*p <* 0.0240) and a significant sex × treatment interaction (*p <* 0.0001) was detected for NLRP3 expression. Accordingly, reserpine administration significantly increased NLRP3 protein levels in the amygdala of both male (*p <* 0.0095; one-way ANOVA vs. VEH; Figure 6C) and female mice (*p <* 0.0055; one-way ANOVA vs. VEH; Figure 6D), whereas no changes were observed in 4-HNE (Figure 6A,B) or p-ERK1/2 expression (Figure 6E,F) in either sex. HRW treatment reversed the reserpine-induced increase in NLRP3 levels in the amygdala of both male and female mice.

### 2.7. The Effect of HRW Treatment on the Expression of HO-1, NQO1 and SOD-1 in the Amygdala of Male and Female Reserpine-Injected Mice

**Figure 7:**
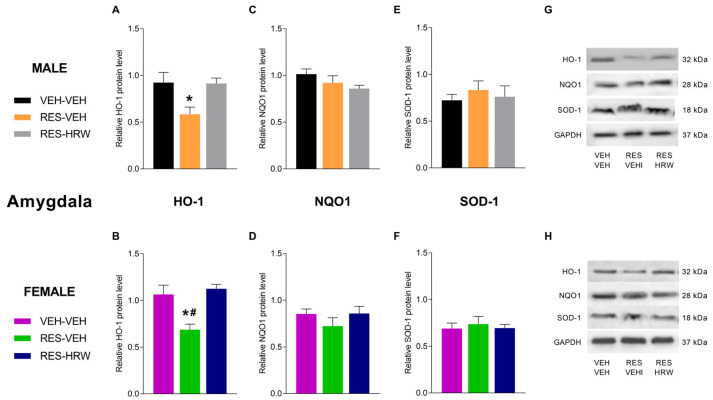
HRW normalizes the antioxidant enzyme expression in the amygdala of reserpine-injected male and female mice. The effects of HRW on the protein levels of heme oxygenase-1 (HO-1; (**A**,**B**)), NAD(P)H quinone dehydrogenase 1 (NQO1; (**C**,**D**)), and superoxide dismutase 1 (SOD-1; (**E**,**F**)) in the amygdala were evaluated in male and female mice injected with reserpine and treated with VEH or HRW. For both sexes, VEH-injected mice treated with VEH are shown as controls. All protein levels are expressed relative to GAPDH. Representative immunoblots for HO-1, NQO1, SOD-1, and GAPDH from male (**G**) and female (**H**) mice are shown. Symbols denote significant differences compared with the corresponding VEH–VEH (*) and RES–HRW (#) groups (*p <* 0.05; one-way ANOVA followed by Sidak’s post hoc test). Tissue samples were collected after six days of HRW treatment (day 28 post-reserpine injection). Data are presented as mean ± SEM (*n* = 4 samples per group). RES, reserpine.

Regarding the effects of HRW on antioxidant enzyme expression in the amygdala, two-way ANOVA revealed a significant main effect of treatment (*p <* 0.0001) on HO-1 protein levels in both male and female mice. Reserpine administration significantly reduced HO-1 expression in the amygdala of male (*p <* 0.0207; one-way ANOVA vs. VEH; Figure 7A) and female mice (*p <* 0.0041; one-way ANOVA vs. VEH; Figure 7B). HRW treatment reversed this reserpine-induced decrease in HO-1 expression in both sexes.

In contrast, reserpine administration did not significantly modify the protein levels of NQO1 (Figure 7C,D) or SOD-1 (Figure 7E,F) in the amygdala of either male or female mice.

## 3. Discussion

Fibromyalgia represents a major clinical challenge, as effective treatments for nociplastic pain frequently accompanied by mood disturbances, particularly depression remain limited. The present study demonstrates that HRW exerts robust antinociceptive and antidepressant-like effects in a reserpine-induced model of fibromyalgia by modulating oxidative stress, neuroinflammatory signaling, and maladaptive plasticity at spinal and/or supraspinal levels, therefore supporting its potential as a therapeutic strategy for fibromyalgia.

In the present study, we employed the reserpine-induced myalgia model, a well-established preclinical paradigm that closely reproduces the core sensory and affective features of fibromyalgia in humans, including mechanical and thermal hypersensitivity, spontaneous pain behaviors, and depressive-like symptoms [17,21,27]. Nevertheless, despite its relevance, few studies have systematically examined sex as a biological variable in this model, as most previous investigations have been conducted exclusively in either male or female animals [17,23,28,29]. Our findings confirm the development of mechanical allodynia, thermal hyperalgesia, and associated depressive-like behaviors previously reported in female reserpine-treated mice and further demonstrate that these alterations are also present in male mice. Importantly, while both sexes exhibited comparable mechanical allodynia and thermal hyperalgesia from days 7 to 28 post-injection, a clear sex-dependent difference emerged in the temporal development of cold allodynia. Cold hypersensitivity appeared earlier in females (day 7) than in males (day 14), although it persisted in both sexes through day 28, coinciding with the emergence of depressive-like behaviors. These findings indicate that sex does not markedly influence the development of mechanical allodynia and thermal hyperalgesia but does modulate sensitivity to cold stimuli. This pattern is consistent with sex-dependent differences reported in other preclinical pain models, including cisplatin-induced chemotherapy-associated neuropathy [30].

HRW treatment progressively reversed mechanical allodynia and thermal hyperalgesia in both sexes, achieving full normalization within four days. In contrast, cold allodynia was abolished after a single day of HRW administration, with a greater inhibitory effect observed in female compared with male mice. This sex-dependent difference suggests that biological factors related to sex may modulate the efficacy of antioxidant-based interventions in nociplastic pain conditions [31]. Similar sex-dependent effects of HRW on cold allodynia have been reported in cisplatin-induced neuropathic pain where females also showed greater responsiveness than males [32]. This heightened sensitivity to cold stimuli and to HRW modulation in females may reflect sex-specific regulation of redox signaling, neuroimmune responses, or hormonal influences on cold-sensitive sensory pathways. Sex differences in antioxidant capacity, mitochondrial function, and neuroimmune signaling have been proposed to contribute to differential vulnerability and recovery in chronic pain conditions [33]. Importantly, HRW did not modify nociceptive responses in control animals, indicating that its antinociceptive effects are specific to pathological pain states rather than reflecting a generalized suppression of sensory processing. This selectivity strengthens the translational relevance of HRW as a potential therapeutic approach.

At the molecular level, reserpine administration induced pronounced oxidative stress in the spinal cord, as evidenced by elevated levels of the lipid peroxidation marker 4-HNE, together with enhanced p-ERK1/2 signaling and upregulation of NLRP3. Both oxidative stress and ERK-dependent synaptic plasticity are well-established contributors to central sensitization and nociplastic pain states [34,35]. Remarkably, HRW normalized spinal 4-HNE levels, in agreement with previous studies demonstrating the antioxidant and neuroprotective properties of H_2_ [36,37]. In parallel, HRW also normalized p-ERK1/2 expression, strongly suggesting that restoration of redox homeostasis and maladaptive synaptic plasticity represents a primary mechanism underlying its antinociceptive effects.

Reserpine also increased spinal NLRP3 expression, supporting the involvement of neuroinflammatory mechanisms in nociplastic pain [38]. However, HRW did not reduce spinal NLRP3 levels, indicating that its analgesic efficacy does not require direct suppression of inflammasome activation. Collectively, these findings suggest that HRW predominantly modulates redox- and plasticity-related pathways that are key drivers of neuronal hyperexcitability and central sensitization in fibromyalgia. Accordingly, symptom improvement in nociplastic pain conditions does not necessarily require complete normalization of inflammatory markers but rather depends on the restoration of redox homeostasis and regulation of neuronal excitability [6].

Importantly, HRW engaged sex-specific antioxidant pathways in the spinal cord, suggesting differential redox strategies underlying its antinociceptive effects. While HRW preferentially upregulated HO-1 in male mice, it selectively increased NQO1 expression in females, indicating distinct modes of antioxidant enzyme activation. In contrast, the reserpine-induced downregulation of SOD-1 was normalized by HRW in both sexes, highlighting a shared basal antioxidant mechanism essential for restoring redox homeostasis and analgesia. Sex-dependent regulation of antioxidant systems has been increasingly recognized in pain and neurodegenerative disorders and may reflect differences in mitochondrial function, hormonal modulation, and redox buffering capacity between males and females [33,39]. These divergent antioxidant responses may contribute to the observed sex differences in cold allodynia, which emerged earlier and was more sensitive to HRW in female mice. Cold hypersensitivity is strongly influenced by redox-sensitive ion channels and spinal excitability, processes that are known to be differentially regulated between sexes [32,40,41].

Beyond nociception, reserpine induced marked depressive-like behaviors in both sexes, reflecting the high comorbidity between chronic pain and mood disorders in fibromyalgia [42,43]. These affective alterations were paralleled by molecular changes in the amygdala, a key hub integrating emotional processing and pain affect [25]. In this region, reserpine downregulated HO-1 and upregulated NLRP3, indicating the presence of oxidative stress and neuroinflammation within supraspinal circuits implicated in depression and pain-related affect. Unlike the spinal cord, HRW fully reversed both HO-1 downregulation and NLRP3 upregulation in the amygdala, suggesting a region-specific sensitivity of neuroimmune mechanisms to molecular hydrogen.

The normalization of amygdala redox balance and inflammasome signaling by HRW provides a plausible mechanistic basis for its antidepressant-like effects. Increasing evidence indicates that NLRP3-driven neuroinflammation in limbic regions contributes to depressive symptoms and affective dysregulation in chronic pain states [11,44]. Thus, HRW may alleviate depressive-like behaviors by restoring neuroimmune homeostasis in the amygdala, while its antinociceptive actions primarily rely on reducing oxidative stress and plasticity at the spinal level. This regional mechanistic dissociation highlights the multifaceted mechanisms through which HRW acts on the pain-depression axis.

The therapeutic effects of HRW observed in the present study are consistent with growing evidence indicating that antioxidant-based interventions can alleviate fibromyalgia-related symptoms. Several commonly consumed bioactive compounds, including capsaicin, ginger, curcumin, omega-3 polyunsaturated fatty acids (n-3 PUFA), grape seed extract, naringin, and genistein, have shown beneficial effects in cellular, animal, and clinical studies of fibromyalgia [45]. In cellular models, these compounds reduce pro-inflammatory mediator production and enhance antioxidant defenses, thereby improving neuronal or myoblast survival and regulating apoptosis-related pathways. In animal models, their administration has been associated with reduced pain hypersensitivity and fatigue as well as improvements in behavioral alterations related to fibromyalgia. These findings support the concept that modulation of oxidative stress and inflammatory pathways represents a key therapeutic strategy in fibromyalgia. In this context, molecular hydrogen represents a promising therapeutic strategy, given its capacity to selectively neutralize highly reactive oxygen species, modulate inflammatory signaling, and rapidly diffuse across biological membranes, thereby restoring homeostasis in both spinal and supraspinal regions involved in pain and affective processing.

Nevertheless, several limitations of this study should be acknowledged. Although redox imbalance was evaluated using well-established oxidative stress markers, direct assessments of mitochondrial function were not performed and could provide additional mechanistic insight. Similarly, inflammasome activation was inferred from changes in NLRP3 expression without measuring downstream cytokine measurements. While the estrous cycle phase was controlled to minimize hormonal variability, future studies examining multiple phases of the cycle may offer a more comprehensive understanding of potential sex-related differences. In addition, HRW was administered intraperitoneally in the present study, which differs from the oral route commonly used in clinical settings. Another limitation is the absence of follow-up assessments after discontinuation of HRW treatment. Consequently, it remains unclear whether the observed antinociceptive and antidepressant-like effects persist or are rapidly reversed once treatment ceases. Addressing the durability of HRW-induced therapeutic effects will therefore be an important objective for future studies. Finally, it should be noted that no single animal model fully reproduces the complexity of human fibromyalgia.

## 4. Materials and Methods

### 4.1. Animals

Male and female C57BL/6 mice (8–10 weeks old; 21–26 g) were obtained from Envigo (Barcelona, Spain). Animals were housed under standard laboratory conditions with a 12 h light/dark cycle, controlled temperature (22 ± 1 °C) and relative humidity (55 ± 10%), and ad libitum access to food and water. Mice were group-housed (4 animals per cage) in polypropylene cages containing wood shavings and maintained in an enriched environment that included a cardboard shelter and cellulose nesting material. The estrous cycle of female mice was monitored by daily vaginal cytology. Behavioral testing and tissue collection were performed during the diestrus phase to minimize hormonal variability.

Experiments were conducted after a seven-day acclimatization period and in accordance with the ethical guidelines of the European Commission Directive 2010/63/EU and Spanish legislation (RD 53/2013). All procedures were approved by the Animal Use and Care Committee of the Autonomous University of Barcelona (ethical approval code: 4581). Every effort was made to minimize animal suffering and to reduce the number of animals used, in compliance with the principles of the 3Rs (replacement, reduction, and refinement).

Male and female mice were randomly allocated to experimental groups using a computer-generated randomization sequence (Microsoft Excel, =RAND()). Sample size was determined a priori using G*Power v3.1.7 software based on pilot data, assuming a two-tailed test (α = 0.05, β = 0.2), resulting in a minimum of six animals per group to detect significant differences in nociceptive and depressive-like behaviors. To comply with the principles of reduction, tissues from behaviorally tested animals were subsequently used for molecular analyses.

Behavioral testing was performed from 9:00 AM to 5:00 PM following a one-week acclimatization period to the housing conditions. Animals were also acclimated to the testing room for 1 h before the commencement of the tests. For each test, the order of animals being tested was randomized daily, and each subject was tested at a different time on each test day. For each animal, two independent investigators were involved: the primary investigator administered the treatment in accordance with the randomization table. This researcher was uniquely cognizant of the treatment group allocation. An additional investigator, blinded to the treatment, evaluated the mechanical allodynia, thermal hyperalgesia, and cold allodynia of these subjects.

### 4.2. Fibromyalgia Induction

Fibromyalgia-like conditions were induced following a previously established protocol [17]. Reserpine was dissolved in acetic acid and diluted in saline solution (0.9% NaCl) to a final concentration containing 0.5% acetic acid. The solution was administered subcutaneously at a dose of 0.25 mg/kg on days 0, 1, 2, 9, 16, and 23. Control animals received subcutaneous injections of the corresponding vehicle (VEH) according to the same administration schedule as the reserpine-treated group.

### 4.3. Nociceptive Tests

Mechanical nociceptive thresholds were assessed using the von Frey filament test (North Coast Medical, San Jose, CA, USA). This method employs a series of calibrated nylon monofilaments of increasing stiffness that exert graded forces when applied to the plantar surface of the paw [46]. Briefly, mice were individually placed in transparent Plexiglas cylinders (20 cm high × 9 cm diameter) positioned on a metal mesh floor. Filaments exerting forces ranging from 0.4 to 3.5 g were applied perpendicularly to the plantar surface, beginning with the 0.4 g filament. The procedure was performed on the hind paws of VEH- and reserpine-treated animals. Depending on the behavioral response, the force of the subsequent filament was decreased or increased following the up–down paradigm. A positive response was defined as paw withdrawal, licking, or shaking. Mechanical withdrawal thresholds were estimated using a curve-fitting method implemented in a custom spreadsheet developed with Microsoft Excel (Microsoft Iberia SRL, Barcelona, Spain).

Thermal hyperalgesia was assessed by measuring paw withdrawal latency in response to a radiant heat stimulus using the plantar test apparatus (Ugo Basile, Varese, Italy). Mice were individually placed in transparent Plexiglas cylinders (20 cm high × 9 cm diameter) positioned on a glass surface and allowed to habituate for 30 min before testing. A focused heat stimulus was then applied to the plantar surface of the hind paw. To prevent tissue damage, a cut-off latency of 12 s was imposed in the absence of a withdrawal response. Paw withdrawal latency was calculated as the mean of three independent trials conducted at appropriate intervals during each testing session.

Cold-induced pain sensitivity was assessed using a cold plate apparatus (Ugo Basile, Varese, Italy). Mice were placed individually on a cold plate maintained at 4 ± 0.5 °C for 5 min, and cold allodynia was quantified by recording the number of hind paw elevations.

All measurements were performed on both hind paws.

### 4.4. Depressive-like Behaviors

Depressive-like behaviors were assessed using the tail suspension test (TST) and the forced swimming test (FST). In the TST, mice were suspended by the tail using adhesive tape placed approximately 1 cm from the tip of the tail and attached to a horizontal bar positioned 35 cm above the floor. Animal behavior was recorded for 8 min, and immobility time was quantified during the final 6 min of the session.

In the FST, mice were individually placed in a Plexiglas cylinder (25 cm high × 10 cm diameter) filled with water (24 ± 1 °C) to a depth of 10 cm. Behavior was recorded for 6 min, and immobility time was analyzed during the last 4 min.

### 4.5. Drugs

Reserpine, acetic acid, and NaCl were obtained from Sigma-Aldrich (St. Louis, MO, USA). HRW was prepared using a hydrogen water generator (Osmo-star, Soriano S.L., Alicante, Spain) based on an electrolysis method to generate H_2_. HRW was freshly prepared immediately before administration to minimize hydrogen loss. The pH of HRW was comparable to that of VEH solutions. HRW was administered intraperitoneally 1 h before behavioral testing at a concentration of 0.3 mM (300 µmol/L), equivalent to 0.3 µmol/mL. The dose was chosen based on previous studies demonstrating its efficacy in models of chronic pain and affective disorders [14,15]. All treatments were injected in a final volume of 10 mL/kg, and control groups received the corresponding VEH at the same volume. Given the high volatility of H_2_, HRW was generated individually for each animal, and immediately injected to minimize gas dissipation.

All drugs were prepared right before their use, and for each group treated with a drug, the corresponding control group received the same volume of the respective VEH. In accordance with previous studies, HRW was intraperitoneally administered at 1 h before the tests and injected in a final volume of 10 mL/kg [14,15].

### 4.6. Experimental Protocol

Experiments were conducted in both male and female mice to evaluate the influence of sex on the effects of HRW in this preclinical fibromyalgia pain model. In the first experimental cohort, mechanical allodynia, thermal hyperalgesia, and cold allodynia were assessed one day before reserpine or VEH administration (day 0) and at 7, 14, 21, and 28 days after the first injection (*n* = 6 per group). In two additional cohorts of mice, depressive-like behaviors induced by reserpine were evaluated at 28 days following the initial injection (*n* = 6 per group).

In the second experimental procedure, the effects of HRW on reserpine-induced mechanical allodynia, thermal hyperalgesia, and cold allodynia were evaluated. Animals received HRW or VEH once daily for six consecutive days, from day 23 to day 28 after the initial reserpine injection. This treatment window was selected to evaluate the therapeutic (rather than preventive) effects of HRW. Nociceptive responses were assessed on days 23, 24, 25, 26, 27, and 28 post-reserpine injection (*n* = 6 per group).

The effects of HRW on reserpine-induced depressive-like behaviors were evaluated in separate cohorts of VEH or reserpine-injected mice. Animals received HRW or VEH once daily for six consecutive days, from day 23 to day 28 after the initial reserpine injection, and depressive-like behaviors were assessed on day 28 (*n* = 6 per group).

Finally, the effects of HRW on the protein expression levels of 4-HNE, NLRP3, phosphorylated ERK1/2 (p-ERK1/2), HO-1, NAD(P)H quinone dehydrogenase 1 (NQO1), and SOD-1 were analyzed in the spinal cord and amygdala of reserpine-injected male and female mice using Western blotting. VEH-injected mice served as controls (*n* = 4 samples per group).

In total, 104 mice were used in this study, comprising 52 males and 52 females.

### 4.7. Western Blotting

Animals were euthanized by cervical dislocation 28 days after the first injection of reserpine or VEH. The lumbar segment of the spinal cord and the amygdala were rapidly dissected, and total protein extracts were prepared. Tissues were homogenized by sonication in a RIPA buffer supplemented with 0.5% protease inhibitor and 1% phosphatase inhibitor cocktail (Sigma-Aldrich, St. Louis, MO, USA). Following sonication, samples were incubated for 1 h at 4 °C to allow protein solubilization and subsequently subjected to a second brief sonication (10 s). Lysates were centrifuged at 700× *g* for 20 min at 4 °C, and the resulting supernatants were collected. Equal amounts of protein (60 µg per lane) were mixed with 4× Laemmli sample buffer, denaturized by heating at 95 °C, and resolved by sodium dodecyl sulfate–polyacrylamide gel electrophoresis (SDS–PAGE) using 4% stacking and 12% resolving gels. Proteins were then electrophoretically transferred onto polyvinylidene fluoride (PVDF) membranes for 90 min. Membranes were blocked for 75 min at room temperature with phosphate-buffered saline containing Tween 20 (PBST) or Tris-buffered saline containing Tween 20 (TBST) supplemented with either 5% non-fat dry milk or 5% bovine serum albumin, as appropriate.

Membranes were incubated overnight at 4 °C with the following primary antibodies: anti-4-HNE; 1:100; Abcam, Cambridge, UK), anti-NLRP3 (1:200; Adipogen Life Sciences, Epalinges, Switzerland), anti-phospho-ERK1/2 (p-ERK1/2; 1:250) and anti-total ERK1/2 (1:250; Cell Signaling Technology, Danvers, MA, USA), anti-HO-1 (1:200; Abclonal Technology, Woburn, MA, USA), anti-NQO1 (1:250; Merck, Billerica, MA, USA), anti-SOD-1 (1:150; Novus Biologicals, Littleton, CO, USA), or anti-glyceraldehyde-3-phosphate dehydrogenase (GAPDH; 1:5000; Merck) as a loading control. After washing, membranes were incubated for 1 h at room temperature with horseradish peroxidase–conjugated anti-rabbit or anti-mouse secondary antibodies (GE Healthcare, Little Chalfont, UK). Immunoreactive bands were visualized using enhanced chemiluminescence reagents (ECL kit; GE Healthcare) and detected with a Chemidoc MP Imaging System (Bio-Rad, Hercules, CA, USA). Band intensities were quantified using ImageJ (version 1.8.0; National Institutes of Health, Bethesda, MD, USA).

### 4.8. Statistical Analysis

Statistical analyses were performed using SPSS version 28 (IBM, Madrid, Spain) and GraphPad Prism version 9.0 (La Jolla, CA, USA). Data are presented as mean ± standard error of the mean (SEM). Normality and homogeneity of variance were assessed using the Shapiro–Wilk and Bartlett’s tests, respectively. A three-way repeated-measures analysis of variance (ANOVA) was applied to evaluate the effects of injection, sex, and time, as well as their interactions, on reserpine-induced nociceptive responses. An additional three-way repeated-measures ANOVA was used to analyze the effects of treatment, sex, and time, and their interactions, on the modulation of nociceptive responses by HRW. For each time point, group differences were further examined using one-way ANOVA followed by Sidak’s post hoc test.

The effects of injection, sex, and treatment on reserpine-induced depressive-like behaviors at 28 days post-injection were analyzed using a three-way ANOVA. Within each sex, group differences were further examined using one-way ANOVA followed by Sidak’s post hoc test. In addition, the effects of HRW on the protein expression levels of 4-HNE, NLRP3, p-ERK1/2, HO-1, NQO1, and SOD-1 in the spinal cord and amygdala of reserpine-injected mice were evaluated using two-way ANOVA, with sex and treatment as factors. When appropriate, group differences were assessed using one-way ANOVA followed by Sidak’s post hoc test.

Statistical significance was set at *p <* 0.05.

## 5. Conclusions

In conclusion, this study demonstrates that HRW effectively reverses both nociceptive and affective alterations in a reserpine-induced model of fibromyalgia in male and female mice. These beneficial effects are associated with normalization of oxidative stress and maladaptive plasticity at the spinal level and restoration of redox and inflammatory homeostasis in the amygdala, highlighting region- and sex-dependent mechanisms underlying pain and depression. The present study further revealed sex-dependent differences in cold pain sensitivity and antioxidant pathway engagement. Taken together, these findings support HRW as a safe and promising therapeutic strategy for nociplastic pain conditions such as fibromyalgia and underscore the importance of targeting central redox and plasticity mechanisms, as well as considering sex as a biological variable, in the development of future treatments.

## Data Availability

All data generated or analyzed during this study are included in this published article.
